# WoZ4U: An Open-Source Wizard-of-Oz Interface for Easy, Efficient and Robust HRI Experiments

**DOI:** 10.3389/frobt.2021.668057

**Published:** 2021-07-14

**Authors:** Finn Rietz, Alexander Sutherland, Suna Bensch, Stefan Wermter, Thomas Hellström

**Affiliations:** ^1^ Department of Informatics, University of Hamburg, Hamburg, Germany; ^2^ Department of Computing Science, Umeå University, Umeå, Sweden

**Keywords:** Human-Robot interaction, HRI experiments, wizard-of-oz, pepper robot, tele-operation interface, open-source software

## Abstract

Wizard-of-Oz experiments play a vital role in Human-Robot Interaction (HRI), as they allow for quick and simple hypothesis testing. Still, a publicly available general tool to conduct such experiments is currently not available in the research community, and researchers often develop and implement their own tools, customized for each individual experiment. Besides being inefficient in terms of programming efforts, this also makes it harder for non-technical researchers to conduct Wizard-of-Oz experiments. In this paper, we present a general and easy-to-use tool for the Pepper robot, one of the most commonly used robots in this context. While we provide the concrete interface for Pepper robots only, the system architecture is independent of the type of robot and can be adapted for other robots. A configuration file, which saves experiment-specific parameters, enables a quick setup for reproducible and repeatable Wizard-of-Oz experiments. A central server provides a graphical interface *via* a browser while handling the mapping of user input to actions on the robot. In our interface, keyboard shortcuts may be assigned to phrases, gestures, and composite behaviors to simplify and speed up control of the robot. The interface is lightweight and independent of the operating system. Our initial tests confirm that the system is functional, flexible, and easy to use. The interface, including source code, is made commonly available, and we hope that it will be useful for researchers with any background who want to conduct HRI experiments.

## 1 Introduction

The multidisciplinary field of Human-Robot Interaction (HRI) becomes more and more relevant, with robots being increasingly present in our everyday life (Yan et al., 2014; Sheridan, 2016). Especially proximate interaction, where humans interact with embodied agents in close proximity (Goodrich and Schultz, 2008), gains increased attention, with social robots taking on different roles as interaction partners. Thus, with social robots becoming more and more integrated and involved in our societies, there is also an increased need to investigate the arising interaction patterns and scenarios. Many such investigations are conducted as “Wizard-of-Oz” (WoZ) experiments, where a human operator (the “wizard”) remotely operates the robot, and controls its movements, speech utterances, gestures, etc.^1^ The test participants interacting with the robot are not aware that the robot is controlled by the wizard. Thus, WoZ experiments allow the investigation of human-robot interaction patterns and scenarios, simulating a robot with advanced functionalities such as using natural language, gaze, and gestures in interaction with a human.

The WoZ paradigm is effective for investigations of hypotheses related to many typical HRI problems. While originally coined and used in human-computer interaction (HCI) research (Kelley, 1984), the WoZ method was introduced as a technique to simulate interaction with a computer system that was more intelligent than currently possible, or practical, to implement. As such, it may be used to study user responses with hypothetical systems, like fluent, real-world interaction with an embodied agent. For example, the WoZ technique was, and still is, commonly used to evaluate and develop dialogue systems (Dahlbäck et al., 1993; Bernsen et al., 2012; Schlögl et al., 2010; Pellegrini et al., 2014). Research in HRI, in particular social robotics, has picked up the idea, and WoZ has, for at least the last 2 decades, been used extensively to investigate the interaction between humans and robots. The possibility to study hypothetical systems is valuable in HRI, in particular when the interaction is unpredictable, and the robot has to adapt to the interacting human to be convincing and engaging.

As Riek shows in a review (Riek, 2012) of 54 research papers in the HRI area, the motivation for, and nature of the usage of WoZ varies widely. However, in 94.4% of the papers, WoZ was used in a clearly specified HRI scenario, whereas in only 24.1% of the papers, WoZ was mentioned as being part of an iterative design process (Dow et al., 2005; Lee et al., 2009). These numbers illustrate how, in the HRI community, WoZ is most often used to study and evaluate the interaction between humans and a given robot, and not as a design tool.

In the HRI community, most WoZ implementations are hand-crafted by research groups, specifically for their own, planned HRI experiments, such as the WoZ system developed and used during the *emote* project^2^. Typically, such systems become problem-specific or experiment-specific and require reprogramming to be used in different experiments. In (Thunberg et al., 2021), a WoZ-based tool for the Furhat robot^3^ for human therapists is presented. The functionality is designed for psychotherapy sessions with older adults suffering from depression in combination with dementia. A graphical user interface allows a therapist to control the Furhat robot’s functionalities, namely speech and facial expressions via configurable clickable buttons and a free-form text box.

A few attempts have been made to develop general WoZ tools for HRI research. The *Polonius* interface from 2011 (Lu and Smart, 2011) is a ROS-based system based on both a Graphical User Interface (GUI) and scripts for configuration and control of robots. While it describes powerful functionality, there seems to be limited continued development of the system. Hoffman (2016) describes *Open-WoZ*, a WoZ framework with largely the same ambition as our system presented in this paper. However, it is unclear how much of the envisioned functionality, e.g. for sequencing of behaviors, is implemented, and whether the software is publicly available. Another example of a recent, publicly available WoZ interface is *WoZ Way*, which provides video and audio streams alongside text-to-speech capabilities (Martelaro and Ju, 2017). While generally promising, this system is tailored for remote WoZ studies in the automotive domain, where experiment participants are engaged in real-world driving, and significant reprogramming of the backend would be required for HRI experiments. Other work address commonly identified problems with WoZ. In (Thellman et al., 2017), techniques to overcome control time delays are suggested using a motion-tracking device to allow the wizards to act as if they were the robot.

To summarize, there is no satisfying, general tool to design and conduct WoZ experiments available in the HRI community. As a consequence, researchers mostly develop and use their own tools, customized for each individual experiment. Besides being inefficient in terms of programming efforts, this also makes it harder for non-technical researchers to conduct WoZ experiments, as they might lack the technical expertise necessary to implement such systems.

This paper presents an architecture for configurable and reusable WoZ interfaces to the HRI community. We provide a concrete implementation of the proposed architecture, denoted *WoZ4U*, on the Pepper robot from SoftBank (Pandey and Gelin, 2018), arguably one of the most used social robots. A short video-demonstration of WoZ4U can be found at https://www.youtube.com/watch?v=BaVpz9ccJQE.

## 2 Methods

To identify the most relevant functionalities for WoZ-based HRI research, we reviewed several HRI publications. Riek’s structured review (Riek, 2012) of the usage of WoZ in HRI provides valuable insight and concludes that the three most common functionalities are natural language processing (e.g. dialogue), nonverbal behavior (e.g. gestures), and navigation.

Many WoZ-based HRI experiments require an interactive multimodal robot (i.e. a robot that processes multiple modalities such as visual and auditory input). In (Hüttenrauch et al., 2006), for example, a WoZ study is performed in which the wizard simulates navigation, speech input and output of the robot. Multimodality in HRI is investigated in WoZ experiments in (Markovich et al., 2019; Sarne-Fleischmann et al., 2017) with a focus on navigation, gesturing, and natural language usage. Interaction using voice and visual cues is investigated in a WoZ study in (Olatunji et al., 2018). The study in (van Maris et al., 2020) uses pre-programmed natural language utterances to facilitate dialogues between a robot and a test participant, but the wizard controls the reactivity of the robot. HRI experiments often require multimodality, and the built-in functionalities are often not sufficiently robust. For example, HRI experiments that use pre-programmed natural language functionalities require the test leaders to control the robot’s responses (Bliek et al., 2020; van Maris et al., 2020) to ensure a natural flow for the human-robot interaction.

Most HRI experiments require a post-analysis, using notes of the wizard’s observations, or video and audio recordings (Aaltonen et al., 2017; Bliek et al., 2020; Singh et al., 2020; Siqueira et al., 2018; Klemmer et al., 2000). The WoZ4U interface supports such post-analysis with the possibility to record visual and auditory data from the perspective of the robot.

**FIGURE 1:**
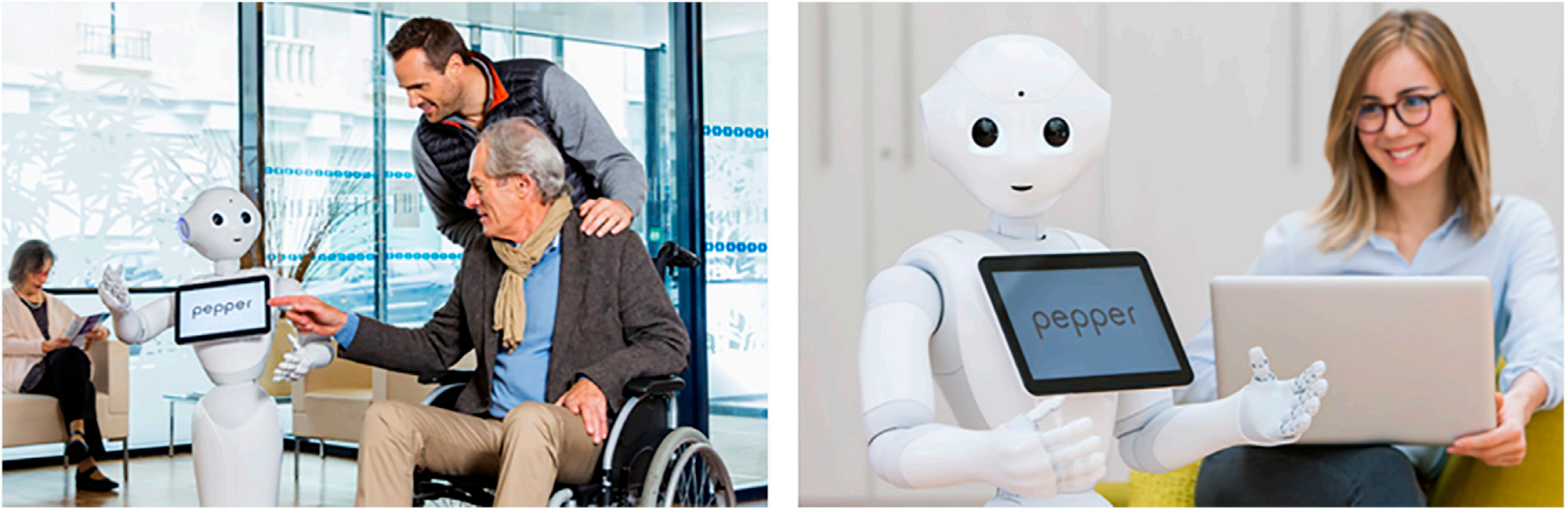
SoftBank’s Pepper robot interacting with humans in different environments (images courtesy of SoftBank Robotics).

Some robots used for HRI experiments allow interaction *via* an integrated touch screen. For example, the android tablet PC on the Pepper robot’s chest (Figure 1) may be used for touch-based input, and to display information such as videos during the experiments (Aaltonen et al., 2017; Tanaka et al., 2015).

To the extent possible, the functionalities mentioned above, identified from other WoZ publications, are included in the developed WoZ4U interface. Table 1 for a summary of the desired and implemented functionalities.

**TABLE 1:** A summary of desired robot functionalities found in the literature, how this is provided in WoZ4U, and the corresponding part number introduced in Figure 3.

Desired functionality	Literature	Implementation in WoZ4U	Part Nr
Documentation of experiments	Aaltonen et al. (2017), van Maris et al. (2020), Bliek et al. (2020), Singh et al. (2020), Siqueira et al. (2018), Markovich et al. (2019), Olatunji et al. (2018)	Recording of data from cameras and microphones	(1)
Supervision of experiments	van Maris et al. (2020), Bliek et al. (2020), Shiomi et al. (2007), Law et al. (2017), Breazeal et al. (2013), Bainbridge et al. (2008)	Real-time monitoring of cameras and microphones	(1)
Accepting non-verbal input	Aaltonen et al. (2017), Tanaka et al. (2015)	Touch interface for the tablet	(1)
Ability to move in the environment	Chapa Sirithunge et al. (2018), Hüttenrauch et al. (2006), Riek (2012), Markovich et al. (2019), Sarne-Fleischmann et al. (2017), Shiomi et al. (2007), Walters et al. (2005)	Control of translation and rotation	(2)
Social cues through facial expressions	Thunberg et al. (2021), Singh et al. (2020), Siqueira et al. (2018)	Control of eyes and LEDs	(6) (7)
Social cues through movements	Andriella et al. (2020), Thunberg et al. (2021), Bliek et al. (2020), Tozadore et al. (2017), Tanaka et al. (2015), Aaltonen et al. (2017), Sarne-Fleischmann et al. (2017)	Gesture control	(8)
Ability to speak in a natural way	Funakoshi et al. (2008), Thunberg et al. (2021), Chapa Sirithunge et al. (2018), Thunberg et al. (2017), Bliek et al. (2020), Tozadore et al. (2017), Singh et al. (2020), Riek (2012), Markovich et al. (2019), Olatunji et al. (2018), Kahn et al. (2008)	Generation of animated speech	(5)
Ability to speak using different voices	Siqueira et al. (2018)	Setting of speech parameters	(5)
Presenting audio-based information	van Maris et al. (2020), Tanaka et al. (2015), Singh et al. (2020), Riek (2012)	Play audio files in loudspeakers	(5)
Presenting image-based information	Tanaka et al. (2015), Bliek et al. (2020), Law et al. (2017)	Display images and videos on tablet	(4)
Varying levels of automated behavior	Aaltonen et al. (2017), Bliek et al. (2020)	Autonomy settings	(3)

### 2.1 System Architecture

**FIGURE 2:**
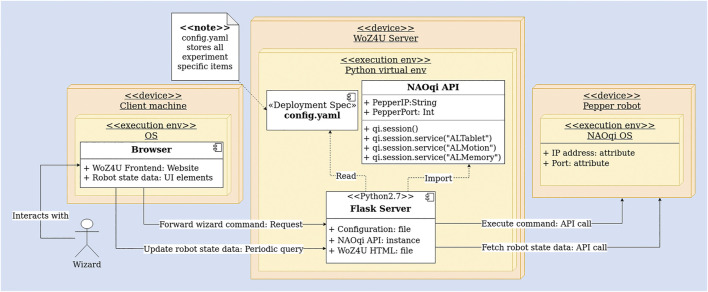
The complete WoZ4U system in the form of an extended deployment UML diagram showing the connected and interacting components, starting with the wizard’s input, and leading to the execution of a command on the robot. The WoZ4U server component maps requests from the frontend to robot API calls and keeps the frontend up-to-date by continuously fetching data from the robot. The frontend is populated with experiment-specific UI elements defined in the configuration file.

The general architecture we present in this paper is based on two ideas. Firstly, we incorporate a configuration file, which holds all experiment-specific items and enables the interface to be adapted easily for different experiments. The configuration file is read during the startup of the system, such that experiment-specific UI elements, as defined in the configuration file, are displayed in the frontend of the system (more on this in Section 2.3). Secondly, we employ a server-based application to implement the control software for the robot. The server, which is implemented with Python’s *Flask* library, provides a graphical user interface (GUI) for the wizard, in the form of one or more web pages. The server handles requests issued by the wizard from the GUI in a browser, and maps them to appropriate robot API calls, such that the robot executes the command instructed by the wizard. In the other direction, the server continuously reads the robotic state (e.g. velocities, ongoing actions, and camera data) from the robot and displays the information in the GUI, such that the interface is always up-to-date. Please refer to Figure 2, illustrating the architecture in an extended deployment UML diagram.

This approach, which displays the robotic state and the control elements in a browser, with a server in the backend, is robot-independent and can be applied to most robotic platforms that provide an API in a programming language capable of running a webserver. This includes all robots supporting the popular Robot Operating System (ROS), due to the *rospy* and *roscpp* libraries. For example, in WoZ Way (Martelaro and Ju, 2017), such an architecture is used as well, and the ROS Javascript library *roslibjs* provides extensive browser support for ROS, which emphasizes the applicability of web-based robot control interfaces. Likewise, the approach to use a configuration file with experiment-specific parameters is robot-independent and may be valuable also for other implementations of this architecture.

The incorporated configuration file makes the system flexible and reusable. To configure a new experiment, only the content of this file has to be modified. In our experience, this works well even for researchers with limited know-how of the lower-level control of the robot. The research on end-user development by Balagtas-Fernandez et al. (2010), Balagtas-Fernandez (2011) supports this, as *configurable components* are proposed as a development tool for non-technical users.

Multimodal and arbitrary complex actions can be defined in the configuration file, which each bind to *one* button in the frontend. This poses a convenient way to lower the cognitive load for the wizard, as more complex and temporally correlated robot behaviors can be achieved through fewer interactions with the frontend.

The distributed nature of the architecture brings several benefits. The server and the frontend do not consume computing resources on the robot. Furthermore, they may run on separate computers, as long as they share the same network as the robot. This enables researchers to use the system without having to install the server on their personal computer, by accessing the frontend via a browser. An additional benefit lies in the availability of the classical HTML, JavaScript, and CSS web development infrastructure, which allows for a very versatile frontend. For example, different views of data sources from the robot can be distributed to additional browser tabs, which can be arranged to fit the user’s specific needs, for example in multiple windows or even on multiple screens. Furthermore, the web development stack is supported by the browser, out-of-the-box, on all modern operating systems, which makes the released system accessible to a wide range of researchers. Lastly, customizing and extending the frontend is straightforward, as editing the HTML-code neither requires recompilation, nor a sophisticated development environment.

In the remainder of this paper, we present a concrete implementation of our architecture, which we refer to as WoZ4U, for SoftBank’s Pepper robot, and describe the implemented functionality.

### 2.2 The Pepper Robot

SoftBank’s Pepper robot was designed with the intent of engaging in social interactions with humans, unlike robots that instead focus on physical work tasks (Pandey and Gelin, 2018). With this goal in mind, the Pepper robot has a humanoid appearance, and a size suitable for interacting indoors with humans in a sitting pose. Figure 1 shows the physical dimension and appearance of the Pepper robot. Pepper is one of the most commonly used research platforms in HRI research, and has also been used in real-world applications, for example, as a greeter in stores (Aaltonen et al., 2017; Niemelä et al., 2017), and as museum guide (Allegra et al., 2018). The Pepper robot has several functionalities required for social HRI. For example, the humanoid design, head-mounted speakers, multicolor LED eyes, and gesturing capabilities allow the robot to express itself in different manners when interacting with humans. The mobile base of the robot allows it to traverse the environment, and the internal microphones and cameras allow Pepper to perceive the world as it engages in social interactions.

The Pepper is usually accessed through the NAOqi Python API^4^ or through Choregraphe (Pot et al., 2009). Using the NAOqi API and Python gives access to a multitude of possibilities, but requires good knowledge of the Pepper software. Choregraphe is a drag-and-drop graphical interface for general programming of the Pepper. It is commonly used to design HRI experiments by programming sequences of interaction patterns such as verbal utterances and gestures issued by Pepper, and corresponding anticipated responses by the test participant. However, Choregraphe is not well-suited when the experimental design requires the robot to respond quickly, adapted to the test participant’s, sometimes unpredictable, behavior. Hence, it is hard to conduct Wizard-of-Oz experiments with the Pepper robot, without investing significant time in programming a suitable control system. With the release of the WoZ4U interface, we hope to significantly lower the threshold for conducting such experiments. We believe that the interface is easier to operate than the two available alternatives (Choregraphe and the Python NAOqi API), especially for researchers without expert knowledge of programming. Our interface provides GUI-based access to functionalities required for HRI experiments, as described in the following.

**Listing 1.** YAML snippet from the configuration file showing how to assign specific NAOqi gestures to buttons in the interface.

**Listing 2.** YAML snippet from the configuration file showing how to define IP addresses for Pepper robots to which the interface can connect.

### 2.3 WoZ4U Graphical User Interface

**FIGURE 3:**
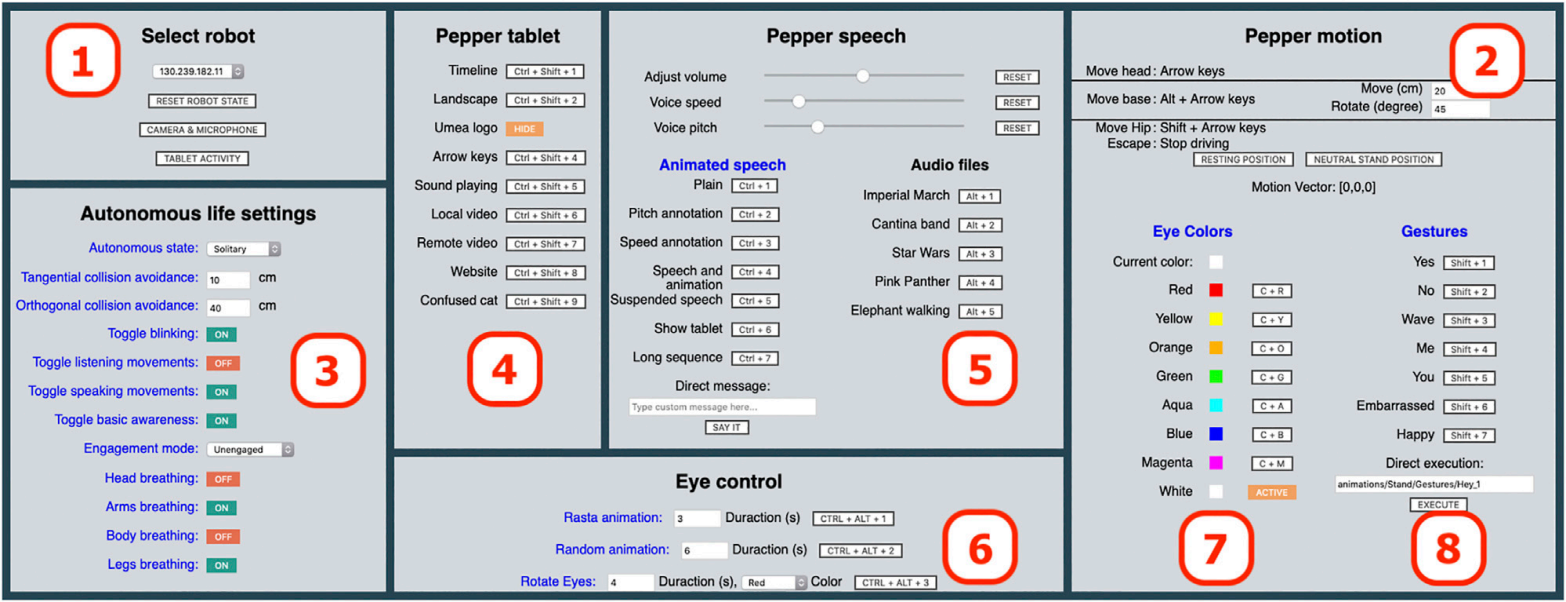
The WoZ4U GUI for Pepper robots, comprising parts (1–8) with the following functionalities: (1) Connection to robot and monitoring, (2) Motion and rotation of head and base, (3) Autonomy configuration, (4) Tablet control, (5) Speech and audio control, (6) and (7) Eye control, and (8) Gesture control.

Figure 3 shows the GUI of the frontend as seen by the wizard. For reference, the GUI in the figure is divided into eight parts labeled **(1–8)** and each part is described in more detail in the following subsections. The wizard can control the robot’s behavior in real-time, either by clicking on buttons using the mouse or by using assigned keyboard shortcuts.

The part headings in the GUI are clickable, and open the documentation of the corresponding part of the API, providing an inexperienced user with more detailed information. To enable tailored design for specific HRI experiments, experiment-specific elements such as keyboard shortcuts, gestures, spoken messages, and image/audio/video file names are defined in a *configuration file* in YAML format^5^. By appropriately modifying this file, the interface will show the commands relevant for the specific experiment, while irrelevant ones are removed.

Multimodal robot behavior can be easily defined in this configuration file. For example, for the Pepper robot to utter “*I am not an intelligent robot. Are you?*” and to gesture at the same time, the configuration file would contain an entry of the form: “I am not an intelligent robot. ${\;}^{\wedge }$ start (gesture) Are you?”.

The configuration file is the only part of the system that has to be modified for a new experiment, and by maintaining multiple files, an experiment may be interrupted and continued later with identical settings. This ensures repeatability between multiple users as well as test participants. Several concrete examples of the configuration file’s syntax and overall structure can be found in the Listings 1, 2, 3 and 4.


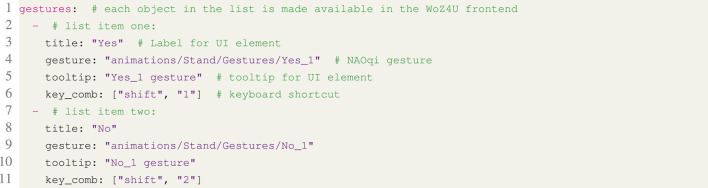



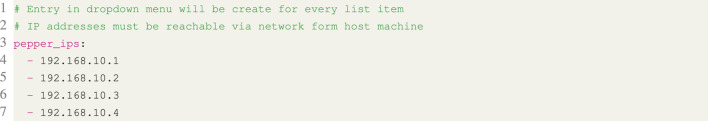



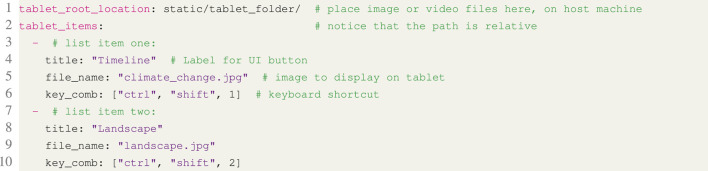



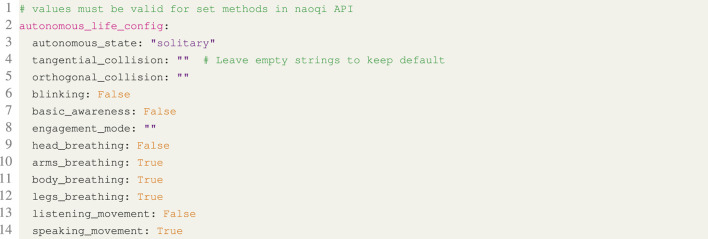

**Listing 3.** YAML snippet from the configuration file showing how to make images available for display on Pepper’s tablet.

**Listing 4.** YAML snippet from the configuration file defining the autonomous life configuration for the Pepper robot.

During the startup of the system, GUI elements are generated based on the given configuration file, and appropriate settings are applied to the robot. Conflicting (duplicated) shortcuts are detected and a warning message is issued.

As many interacting processes are running in the background on the Pepper, especially regarding autonomous life functionality, settings or states on the robot may change automatically, without any commands being issued by the wizard. This may, for example, happen when Pepper switches between its available interaction modes, depending on detected stimuli in its environment. To ensure that the state of the Pepper displayed in the browser (as seen by the wizard) and the actual state of the physical Pepper are the same, the NAOqi API is continuously queried and in case of a discrepancy between the interface and the actual physical state, the GUI is updated accordingly.

#### 2.3.1 Connection to Robot and Monitoring [Part (1)]

After the startup process has finished, the Pepper robot must be connected by selecting the robot’s IP address from the drop-down menu in Part (1) (Figure 3). The IP address (es) must have been previously defined in the configuration file. Note that the computer must be in the same WiFi network as the Pepper robot, such that the IP address of the robot is reachable via TCP/IP. Once the connection with the robot has been established, the GUI is ready to use.

The drop-down menu provides easy access to different robot IP addresses to support several wizards operating several robots (i.e. robot teams) in human-robot interactions.

Part (1) also contains controls to provide access to Pepper’s camera and microphone data. If activated, a new browser tab with additional on/off controls is opened. To accomplish the transmission of microphone data, we extend the NAOqi API, which does not include the necessary functionality. By pressing the button “TABLET ACTIVITY”, an additional browser tab is opened, with information on touch events from the Pepper’s tablet. This enables the wizard to easily monitor touch-interaction on the tablet. The distribution of functionality to dedicated browser tabs prevents overloading the main GUI tab, and also makes it possible to rearrange the screen contents, for example, by dragging tabs to separate windows or even to a secondary screen.

#### 2.3.2 Motion and Rotation of Head and Base [Part (2)]

Part (2) of the GUI is used to move the robot using predefined keyboard shortcuts:

• Arrow keys (alone): Rotate Pepper’s head around the pitch axis (up/down) and yaw axes (left/right).
• Alt + Arrow keys: With a given increment, drive forward or backward, or rotate around the robot’s vertical axis.
• Shift + Arrow keys: With a given increment, rotate the robot’s hip around the pitch (forwards/backward) and roll (sideways) axes.
• Esc: Emergency break, immediately stops all drive-related movements.

The increments for rotation and motion are pre-defined and may be altered in the GUI.

#### 2.3.3 Autonomy Configuration [Part (3)]

Part (3) of the GUI addresses the autonomous life settings of the Pepper robot. These settings are important for most HRI experiments since they govern how the robot reacts to stimuli in the environment and how it generally behaves. The settings often influence each other, sometimes in a non-obvious manner. For example, even if the blinking setting is toggled on, Pepper only blinks when its autonomous configuration is set to “interactive”. In this case, the setting is enabled and displayed correctly but is ignored at a lower level in the NAOqi API. To tackle such non-intuitive behavior, the wizard is informed (by a raised Javascript alert) whenever conflicting settings are made. Pepper’s *autonomous state*, which is displayed in Part (3) of the GUI, is affected by independent Pepper functionalities that sometimes override the API. Hence, observed sudden changes in the state may arise. While it would be possible to enforce Pepper to never diverge from the state specified in the configuration file, this would cause unintended behavior and conflicts with how the NAOqi API is meant to be used.

#### 2.3.4 Tablet Control [Part (4)]

Part (4) of the GUI provides access to Pepper’s tablet. File names of videos and images or URLs to websites can be defined in the configuration file, which can then be shown on the tablet, for example by pressing the designated keyboard shortcut. Showing images on Pepper’s tablet and monitoring touch events on the tablet (as described in 2.3.1) can be a powerful, additional mode for interaction with experiment participants. Displaying websites may, for example, allow participants to fill out forms or answer queries. It is possible to flag a specific image as the default image in the configuration file. This causes the image to be displayed on the tablet once the connection to a Pepper robot is established. Further, when no other tablet contents are currently being displayed, the systems will fall back to and display the default image on Pepper’s tablet. If no image is flagged as default, Pepper will display its default animation on the tablet when no other tablet contents are set active.

#### 2.3.5 Speech and Audio Control [Part (5)]

Part (5) addresses the speech and audio-related functionality of the Pepper robot. Sliders for volume, voice pitch, and voice speed are provided. Text messages and associated keyboard shortcuts may be predefined in the configuration file. The messages may be plain text or contain tags defined in the NAOqi animated speech module^6^. Table 2 lists tags that change parameters of the speech module. Tags may also be used to execute gestures alongside a spoken message, as shown in Table 3. This makes it possible to define convenient combinations of speech and gestures, that can be activated by one keyboard shortcut. For example, instead of first telling the participant to look at Pepper’s tablet, and then executing a gesture where Pepper points at its tablet, a tagged string can accomplish the same thing.

**TABLE 2:** Tags that can be included in sentences to be spoken by the robot. The range and default values are standardized scales provided by SoftBank.

Tag	Function	Range	Default
\\vct = value\\	Changes the pitch of the voice	50–200	100
\\rspd = value\\	Changes the speaking rate	50–400	100
\\pau = value\\	Pauses speech for value msec	—	—
\\vol = value\\	Changes the volume for speech	0–100^7^	80
\\rst\\	Resets control sequences to default	—	—

**TABLE 3:** Control sequences for execution of animations that may be mixed with regular text in sentences.

Control sequence	Function
^run(A)	Suspend speech, run animation A, resume speech
^start(A)	Start animation A (speech is uninterrupted)
^stop(A)	Stop animation A
^wait(A)	Suspend speech, run animation A until it is finished, then resume speech

Apart from the text messages, audio files can be played *via* Pepper’s speakers. Due to strong limitations in the NAOqi API, this requires additional preparation beyond the configuration file. Concretely, the audio files (preferably.wav) must be stored on the Pepper robot directly. After storing the audio files on the Pepper, the absolutes paths to those files (on the Pepper), must be provided in the configuration file. This is because the only reliable way to play sound files on Pepper’s speaker is to play files that are stored directly on the Pepper. While other solutions are possible in theory (e.g. hosting them remotely), we found them to be unreliable and limiting other API calls, and thus opt for this restrictive solution.

#### 2.3.6 Eye Control [Part (6) and Part (7)]

Part (6) and Part (7) of the GUI provide control over Pepper’s RBG eye LEDs. Part (7) controls a configurable set of colors, which can be assigned to Pepper’s eye LEDs, either via button clicks or keyboard shortcuts. Additionally, the available colors and keyboard shortcuts assigned to each color are configurable. Similar to the tablet items, described in Section 2.3.4, a color can be defined as the default color, which will then be applied to Pepper’s LEDs, once a connection to a robot is established. The current color of the eyes is indicated by a colored rectangle in the GUI. This is sometimes helpful since some Pepper gestures change the color of the LEDs. Part (6) of the GUI provides access to the few animations for the eye LEDs that come with the NAOqi API. Since there are only three animations available in total, it is not possible to further configure which animations are accessible. Instead, the default duration for the animations can be stored in the configuration file. The animation *Rotate Eyes*, which lights the eye LEDs in a rotating manner, requires a color code as an argument. The color for the rotation can be selected from a drop-down menu that contains the same colors that are predefined for access in the configuration file.

#### 2.3.7 Gesture Control [Part (8)]

**FIGURE 4:**
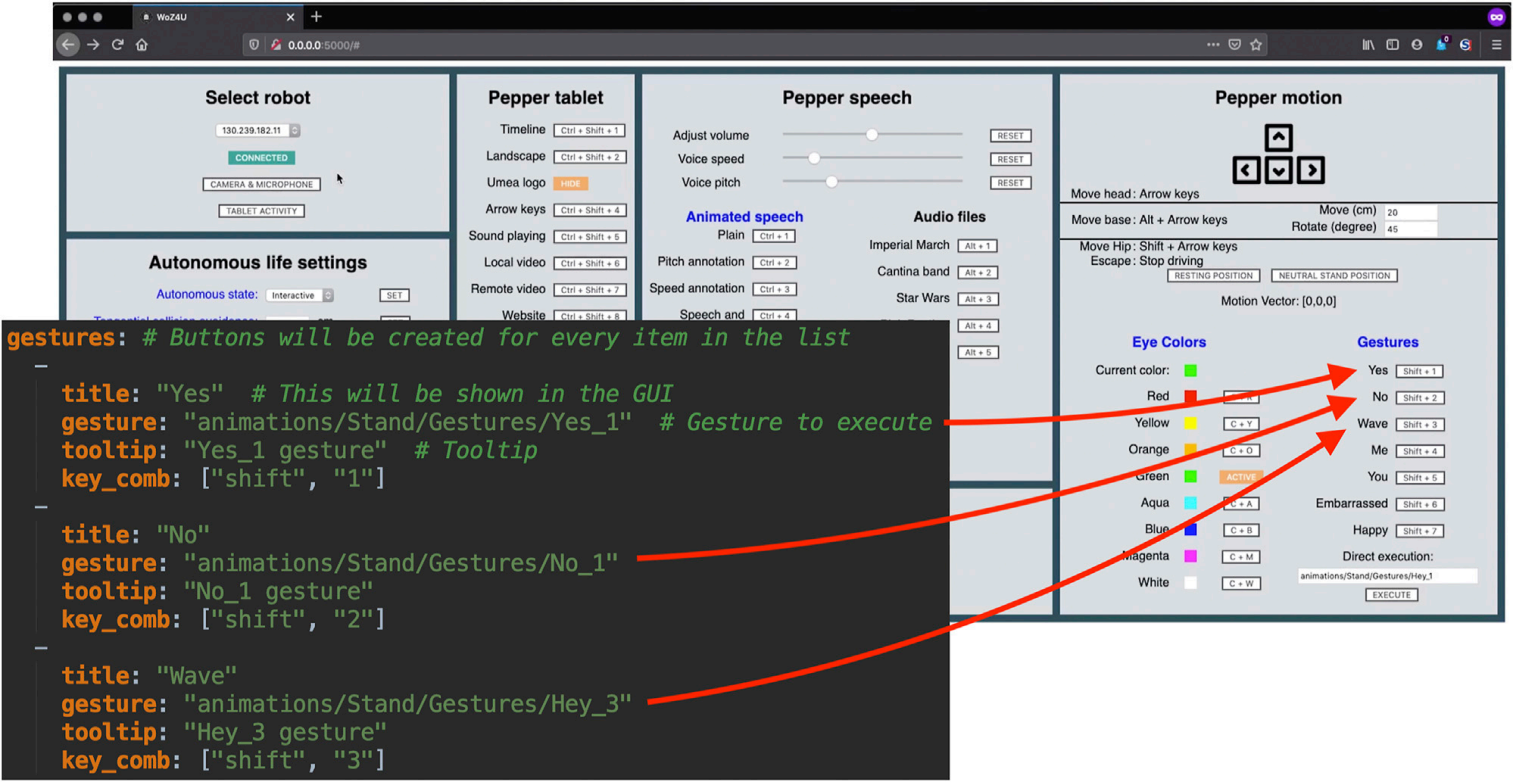
Illustration of how items in the configuration file control the appearance of the GUI.

The Pepper is equipped with a large number of predefined gestures that, when executed, move the head, body, and arms of the robot in a way that resembles human motions associated with greetings, surprise, fear, happiness, etc. Such gestures may be executed on demand by the wizard using keyboard shortcuts. In the configuration file, gestures (defined through their path names) are assigned to keyboard shortcuts, and each gesture is also given a name that is displayed in Part (8) of the GUI. Figure 4 shows an example of how gestures may be specified in the configuration file. Keyboard shortcuts are defined with flags of the form key_comb:[keycode_0,keycode_1].

## 3 Results

This paper presents WoZ4U, a configurable interface for WoZ experiments with Softbank’s Pepper robot. The interface provides utilization, monitoring, and analysis of multiple input and output modalities (e.g. gestures, speech, and navigation). The work of setting up HRI experiments is reduced from programming a complete control system to adjusting experiment-specific items in a configuration file.

Reproducibility of experiments and repeatability between multiple users, as well as test participants, is highly important in HRI, and in research in general (Plesser, 2017). These aspects are also supported by the configuration file approach since configurations for different experiments can be easily maintained and shared between researchers and research communities. As a comparison, in the WoZ Way interface (Martelaro and Ju, 2017), questions are defined in HTML code, and non-trivial rewriting and testing is required for each experiment.

While we do not include an extensive user study in this article, we let four users U1-U4 install and use the WoZ4U in real experiments. U1 and U3 had a technical background (i.e. robotics, programming skills) whereas U2 and U4 had a non-technical background (i.e. interaction and design, social robotics). The users used the WoZ4U interface for different purposes, and controlled speech output, movement, head movements, and gestures. One wizard controlled a robot interacting with a human during a board game displayed on the chest-mounted tablet for about 25 min. The wizard could see and hear the human through the interface, and reacted effortlessly also to unpredictable events (e.g. the human’s actions or network problems). Three of the users acted as wizards in a human-robot team scenario and effortlessly controlled the robot for about 2 h including shorter breaks.

After using the interface, the users filled in a simple questionnaire with eight statements Q1-Q8. The results are summarized in Table 4. For statements Q1-Q7, the answers were one of “Strongly disagree”, “Disagree”, “Neutral”, “Agree”, “Strongly Agree”, coded as 1,2,3,4,5 in the table. Q8 was answered by a number between 1 (very bad) and 10 (very good). The results suggest that the installation procedure could be made more user-friendly (Q1, Q2). However, once installed, the system was quickly understood (Q4), both regarding control (Q3, Q7) and configuration (Q6). The overall usability was rated high (Q8), and WoZ4U was mostly preferred over alternative tools (Q5).

**TABLE 4:** Results from questionnaire answered by four users U1-U4.

Question	U1	U2	U3	U4
Q1: It didn’t take me long to install the system	2	5	3	2
Q2: I think people with limited technical know-how could follow the installation guide	2	5	2	4
Q3: The visual structure of the interface is coherent and supportive with respect to operating Pepper	4	5	4	4
Q4: It didn’t need a lot of practice to use the tool	4	5	5	4
Q5: I prefer using WoZ4U over alternative systems to control Pepper	4	5	3	3
Q6: I quickly understood how to edit the configuration file to change the elements in the interface	4	5	3	5
Q7: The buttons and other controls produced predictable results on the robot	3	5	4	4
Q8: Rate the overall usability (“ease of use”) of the WoZ4U system	7	9	6	8

Overall, our assessment of the four wizards’ behavior, combined with the results from the questionnaire, supports our belief that WoZ4U is a flexible and useful tool, for both technical and non-technical researchers.

Since both Python and the NAOqi API are available on all common modern operating systems (e.g. Debian-based Linux, Mac OS, and Windows), WoZ4U can be installed on all these systems.

Besides providing a WoZ interface for the Pepper robot, we describe the general architecture of the system. The publicly available source code may serve as a starting point for implementations on other robot platforms. The source code may also be useful when developing other programs for the Pepper robot, since the official documentation lacks code examples for several parts of Pepper’s API.

## 4 Discussion

The WoZ4U interface may be further developed in several respects. As argued in (Martelaro, 2016), a WoZ interface may, or even should, include not only control functionality but also display data from the robot’s sensing and perception mechanisms. This is particularly valuable if the WoZ experiment is a design tool for autonomous robot functionality. For example, a robot’s estimation of the test participant’s emotion (based on camera data) may be valuable to monitor, in order to assess the quality of the estimation, and also as a way to adapt the interaction with the participant, in response to a possibly varying quality of the estimation during the experiment. However, such features would be experiment-specific and are not appropriate for a general-purpose WoZ tool. Nevertheless, such functionality can be easily added in additional browser tabs, in the same way as already done for the camera and audio data (Part 1).

The usage of WoZ in HRI is sometimes criticized for turning an experiment into a study of human-human interaction rather than of human-robot interaction (Weiss et al., 2009). One aspect of this problem is related to the fact that the wizard often acts on perceptual information at a higher level than an autonomous robot would do. One approach to prevent this is to restrict the wizard’s perception (Sequeira et al., 2016). Another aspect is investigated in (Schlögl et al., 2010), where reported experiments show how the wizard’s behavior sometimes varies significantly, thereby potentially influencing the outcome of the experiment. Sometimes, this can be avoided with a non-WoZ approach, i.e. by programming the robot to interact autonomously with the test participants (Bliek et al., 2020). Another approach, which addresses both aspects mentioned above, is to automate and streamline the wizard’s work as much as possible. WoZ4U provides keyboard shortcuts and other functionalities towards these ends, but this could be further advanced by introducing more complex, autonomous robot behaviors. For example, by supporting the combination of multiple gestures into larger chains of interaction blocks, even larger chunks of the interaction could be automated. This would not only simplify the wizard’s work but also ensure a more consistent and human-robot-like interaction. More such advancements could be guided by an empirical usability study of the WoZ4U interface, alongside a cognitive load analysis of wizards using the system.

The evaluation of the system showed that the installation instructions provided for the WoZ4U system may be hard to follow for non-technical users. Hence, an automated installation solution would greatly benefit non-technical users and make the system as a whole even more accessible.

## Data Availability

WoZ4U is available under the MIT license, and downloadable at https://github.com/frietz58/WoZ4U. This web page also provides additional information on how to install WoZ4U, and how to write and modify configuration files.

## Footnotes

1 Sometimes several wizards control the same robot, as a way to provide real-time responses by the robot, see, for example, (Marge et al., 2016).

2
http://gaips.inesc-id.pt/emote/woz-wizard-of-oz-interface/.

3
https://furhatrobotics.com/.

4
http://doc.aldebaran.com/2-5/naoqi/index.html.

5
https://yaml.org/.

6
http://doc.aldebaran.com/2-5/naoqi/audio/alanimatedspeech.html.
